# Supplementary material for: Selective Inhibition of Deamidated Triosephosphate Isomerase by Disulfiram, Curcumin, and Sodium Dichloroacetate: Synergistic Therapeutic Strategies for T-Cell Acute Lymphoblastic Leukemia in Jurkat Cells
Source: Biomolecules. 2024 Oct 13;14(10):1295. doi: 10.3390/biom14101295 (PMC11506356; doi:10.3390/biom14101295)
Supplement: Supplementary file 1 [file biomolecules-14-01295-s001.zip › biomolecules-3202107-supplementary.pdf]

Type of the Paper (Article)

Selective inhibition of deamidated triosephosphate isomerase by disulfiram, curcumin, and sodium dichloroacetate: Synergistic therapeutic strategies for T-cell acute lymphoblastic leukemia in Jurkat cells.

Luis Antonio Flores-López [1], Ignacio De la Mora-De la Mora [2], Claudia M. Malagón-Reyes [3], Itzhel García-Torres [2], Yoalli Martínez-Pérez [4], Gabriela López-Herrera [5], Gloria Hernández-Alcántara [6], Gloria León-Avila [7], Gabriel López-Velázquez [2], Alberto Olaya-Vargas [8], Saúl Gómez-Manzo [9] and Sergio Enríquez-Flores [2,*]

[1] Laboratorio de Biomoléculas y Salud Infantil, CONAHCYT-Instituto Nacional de Pediatría, Mexico City 04530, Mexico.
[2] Laboratorio de Biomoléculas y Salud Infantil, Instituto Nacional de Pediatría, Mexico City 04530, Mexico; ignaciodelamora@ciencias.unam.mx (I.D.l.M.-D.l.M.); garciaitzhel@gmail.com (I.G.-T.); glv_1999@ciencias.unam.mx (G.L.-V.)
[3] Posgrado en Ciencias Biológicas, (Maestría), Universidad Nacional Autónoma de México, Mexico City 04510, Mexico; cmmalagonr@gmail.com
[4] Instituto Tecnológico y de Estudios Superiores de Monterrey, Campus Ciudad de México, Mexico City 14380, Mexico; yoalli.martinez@tec.mx
[5] Laboratorio de Inmunodeficiencias, Instituto Nacional de Pediatría, Mexico City 04530, Mexico; lohegabyqbp@gmail.com
[6] Departamento de Bioquímica, Facultad de Medicina, Universidad Nacional Autónoma de México, Apartado Postal 70-159, Mexico City 04510, Mexico; ghernandez@bq.unam.mx
[7] Departamento de Zoología, Escuela Nacional de Ciencias Biológicas, Instituto Politécnico Nacional, Carpio y Plan de Ayala S/N, Casco de Santo Tomás, Ciudad de México 11340, Mexico; leonavila60@yahoo.com.mx
[8] Trasplante de Células Madre y Terapia Celular, Instituto Nacional de Pediatría, Mexico City 04530, Mexico; alberto.olaya@yahoo.com.mx
[9] Laboratorio de Bioquímica Genética, Instituto Nacional de Pediatría, Mexico City 04530, Mexico; saulmanzo@ciencias.unam.mx
* Correspondence: luisbiolexp@gmail.com (L.A.F.-L.); sergioenriquez@ciencias.unam.mx (S.E.-F.)

Supplementary Table S1. List of primary and secondary antibodies used in western blots in this study.

Primary antibody	Catalogue	Secondary antibody	Provider	Dilution
TIM antibody (H-11)	sc-166785	Mouse	Santa Cruz Biotech	1:1000
ERK 1/2 (C-9)	sc-514302	Mouse	Santa Cruz Biotech	1:1000
p-ERK1/2 (12D4)	sc-81492	Mouse	Santa Cruz Biotech	1:1000
Bcl-2 (C-2)	sc-7382	Mouse	Santa Cruz Biotech	1:1000
Bax (B-9)	sc-7480	Mouse	Santa Cruz Biotech	1:1000
Caspase-7 (10-1-62)	sc-56063	Mouse	Santa Cruz Biotech	1:1000
β-Actin (C-2)	sc-8432	Mouse	Santa Cruz Biotech	1:1000

For detection, a horseradish peroxidase (HRP)-conjugated anti-mouse IgG H&L secondary antibody, ab6728 (Abcam, Cambridge, UK), diluted 1:3000, was used.

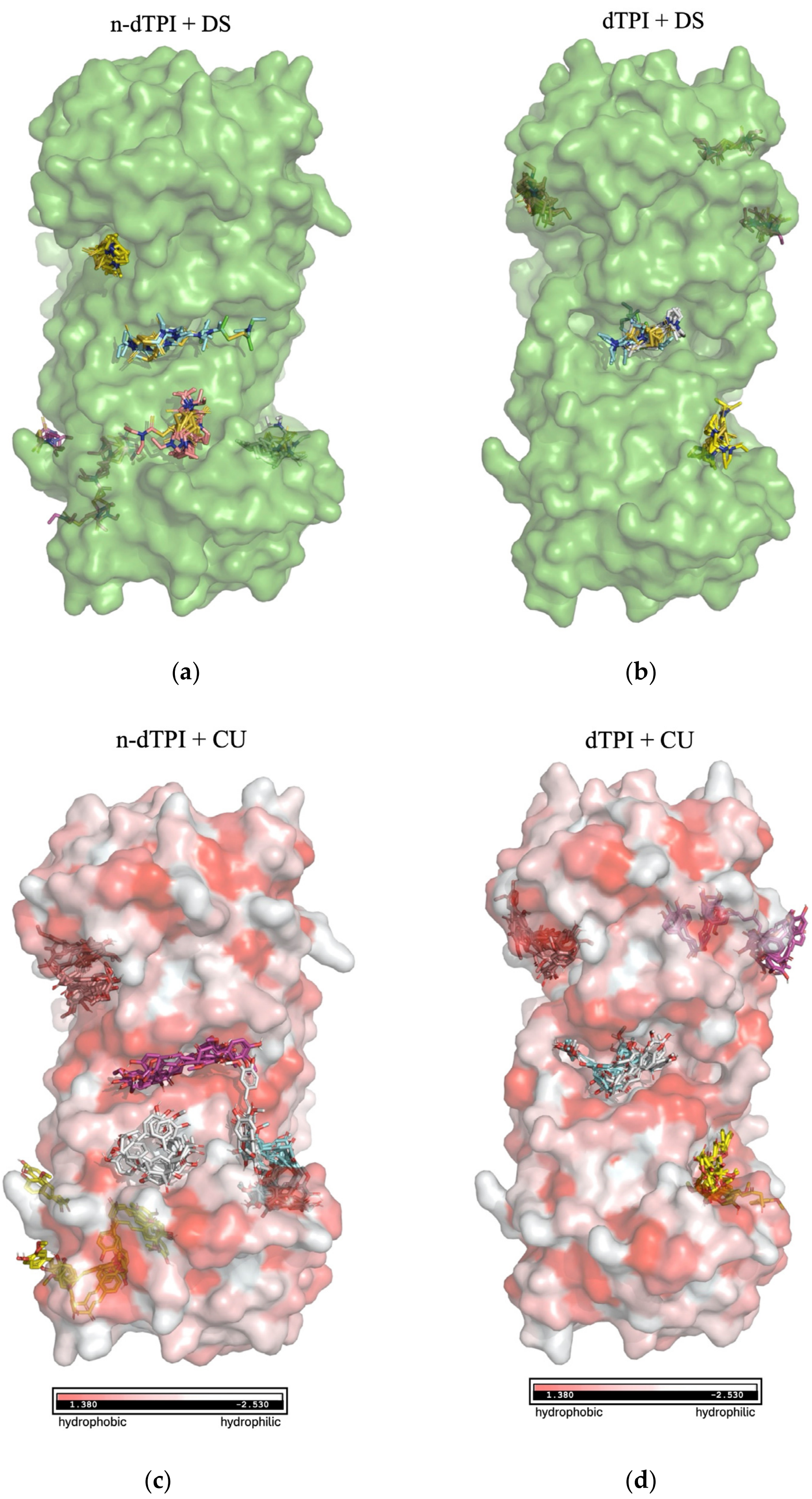

Supplementary Figure S1. Molecular docking analysis of n-dTPI and dTPI crystallographic structures. The docking sites of DS in n-dTPI and dTPI are depicted, with panels **a** and **b** showing the distinct cavities where DS was docked along with the corresponding conformers. Similarly, panels **c** and **d** display the different cavities where CU was docked, along with the respective conformers for each cavity.

Supplementary Table S2. Predicted binding affinities of DS to pockets in n-dTPI and dTPI by docking Simulations.

Pocket (n-dTPI) (PDB ID: 2jk2)	Docking Score (kcal/mol)	Pocket (dTPI) (PDB ID: 4unk)	Docking Score (kcal/mol)
[1] 1	-3.2	[1] 1	-4.3
2	-3.2	2	-4.3
3	-3.1	3	-4.3
4	-3.1	4	-4.3
5	-3.0	5	-4.3
6	-3.0	6	-4.3
7	-2.9	7	-4.2
8	-2.9	8	-4.2
9	-2.9	9	-4.0

[1] Pocket located on the protein interface.

Supplementary Table S3. Predicted binding affinities of CU to pockets in n-dTPI and dTPI by docking Simulations.

Pocket (n-dTPI) (PDB ID: 2jk2)	Docking Score (kcal/mol)	Pocket (dTPI) (PDB ID: 4unk)	Docking Score (kcal/mol)
[1] 1	-7.8	[1] 1	-8.9
2	-7.4	2	-8.7
3	-7.2	3	-8.5
4	-7.1	4	-8.0
5	-7.0	5	-7.8
6	-6.5	6	-7.8
7	-6.3	7	-7.6
8	-6.3	8	-7.4
9	-6.2	9	-7.3

[1] Pocket located on the protein interface.

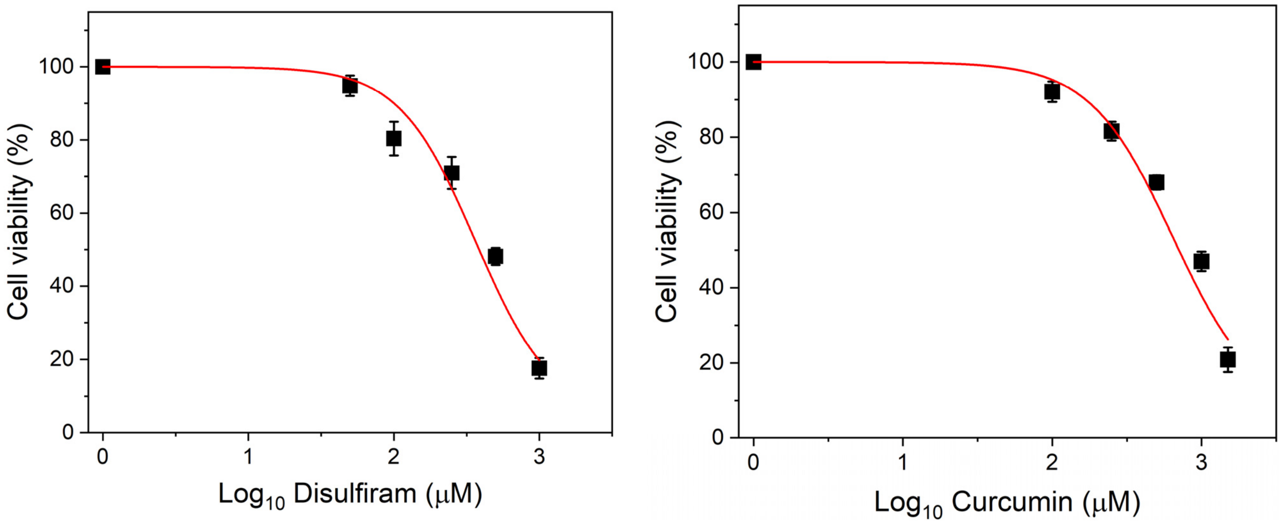

(**a**) (**b**)

Supplementary Figure S2. IC_{50} determination in Jurkat cells treated with CU or DS. Semilogarithmic plot of concentration *vs* cell viability. The fit (red line) represents a three-parameter logistic regression model. Results are expressed as percentages relative to the untreated control group (set at 100%). Data represent the mean of three independent experiments, with error bars indicating the variability across experiments.

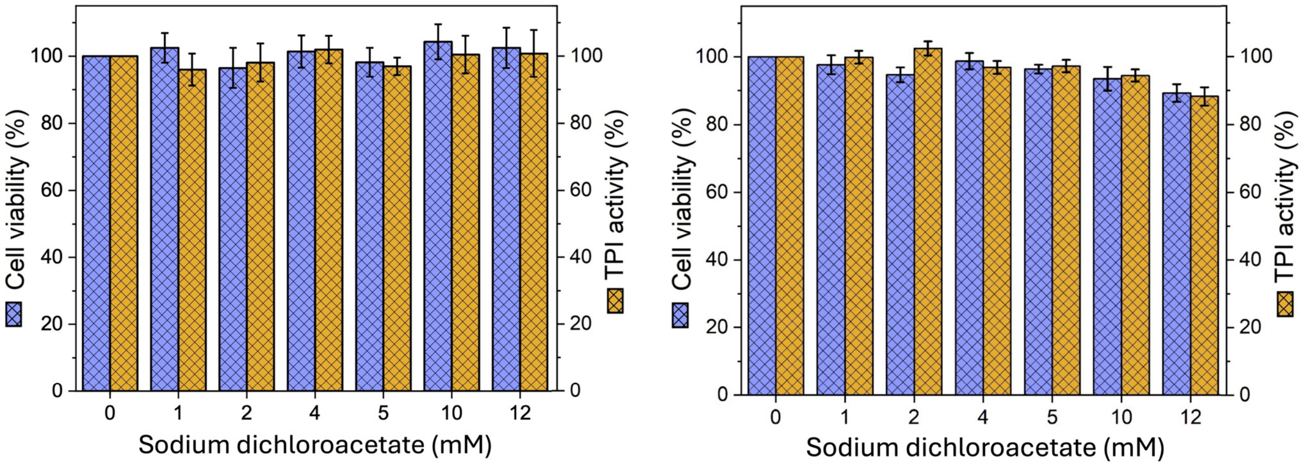

(**a**) (**b**)

Supplementary Figure S3. Effects of DCA on cell viability and TPI activity in normal T lymphocytes and Jurkat cells. Cells (1 x 10^5 per well) were incubated with increasing concentrations of DCA. Following incubation, cell viability was assessed using MTT assays, and TPI activity was determined by enzymatic activity assays. (**a**) normal cells, (**b**) cancer cells. Results are expressed as percentages relative to the untreated control group (set to 100%). Data represent the mean ± SD of three independent experiments.

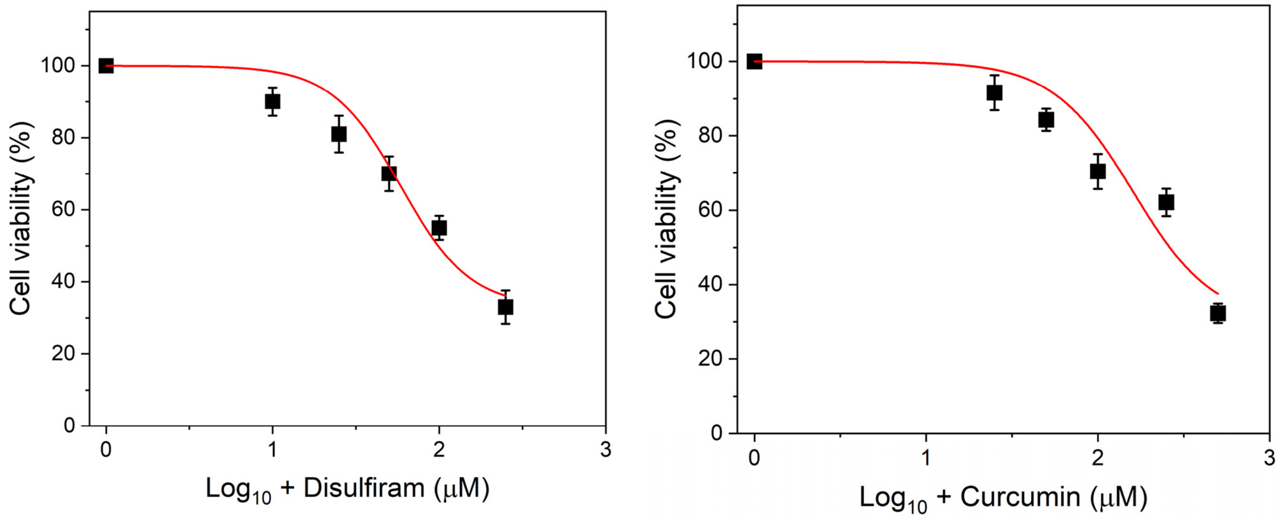

(**a**) (**b**)

Supplementary Figure S4. IC$_{50}$ determination in Jurkat cells pre-treated with DCA and subsequently treated with a combination of DS or CU. Semilogarithmic plot of concentration *vs* cell viability. The fit (red line) represents a three-parameter logistic regression model. Results are expressed as percentages relative to the untreated control group (set at 100%). Data represent the mean of three independent experiments, with error bars indicating the variability across experiments.

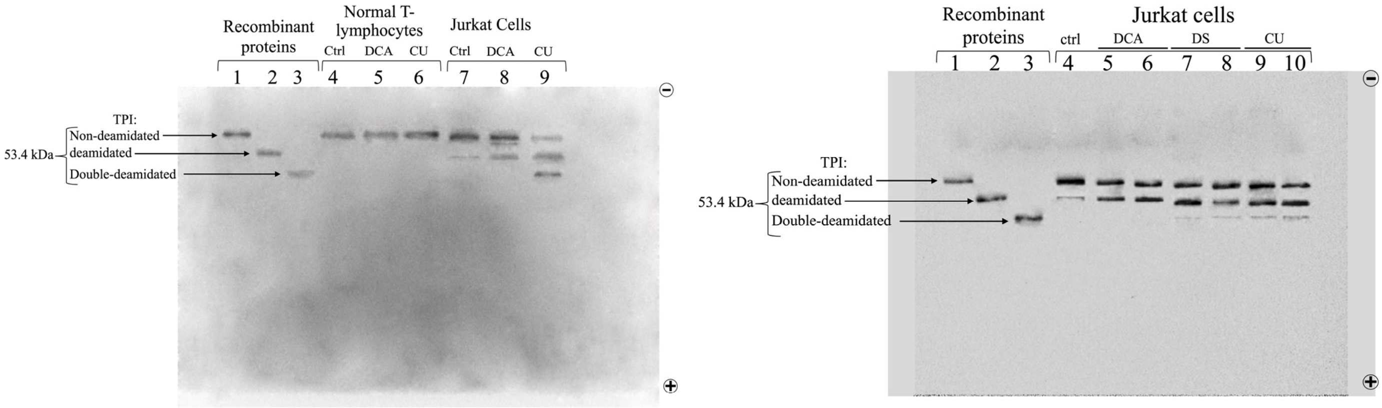

(**a**) (**b**)

Supplementary Figure S5. Full-length of western blots of Figure 6. Panels **a** and **b** correspond to the blots from nPAGE of recombinant and cellular TPIs; the arrows indicate the acidic species of TPIs. The positive and negative poles of the gel are indicated on the right side of each panel. In nPAGE, proteins migrate according to their charge-to-mass ratio. The TPI monomer has a molecular weight of approximately 26.7 kDa. Since TPI typically exists in a dimeric form under native conditions, it migrates with an apparent molecular weight of around 53.4 kDa, corresponding to its native oligomeric state.

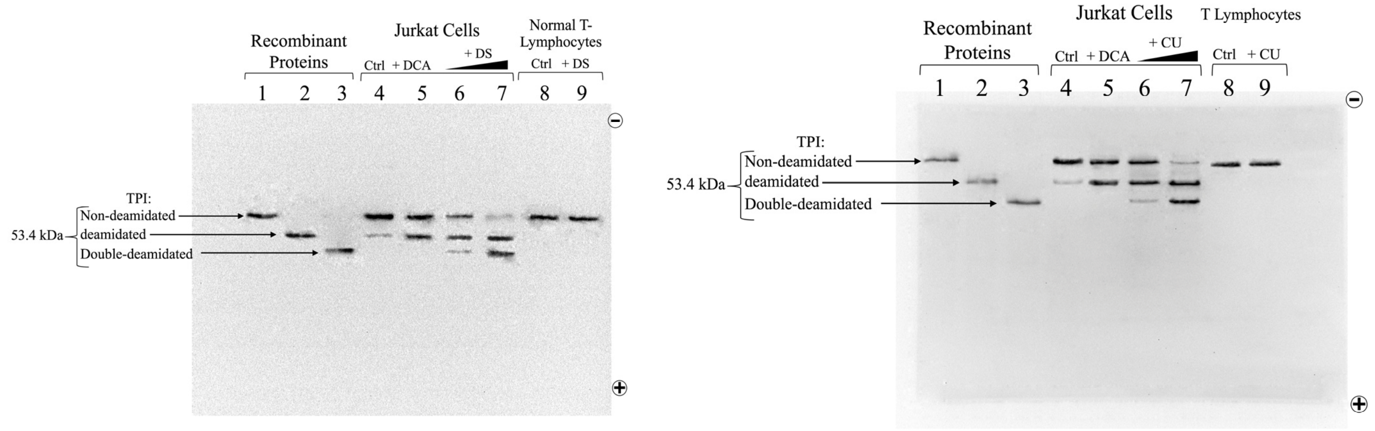

(**a**) (**b**)

Supplementary Figure S6. Full-length of western blot of Figure 7. Uncropped blot from nPAGE of recombinant and cellular TPIs; the arrows indicate the acidic species of TPIs. The positive and negative poles of the gel are indicated on the right side of each panel. The TPI monomer has a molecular weight of approximately 26.7 kDa. Since TPI typically exists in a dimeric form under native conditions, it migrates with an apparent molecular weight of around 53.4 kDa, corresponding to its native oligomeric state.

Supplementary Table S4. Relative abundance of TPI isoforms in Jurkat cells as illustrated in Figures 6 and 7.

Condition	n-dTPI (%)	dTPI (%)	ddTPI (%)
**No treatment**	100	10	[1]Nd
**12 mM DCA**	100	90.5	[1]Nd
**250 μM DS**	100	90	10
**1500 μM CU**	100	150	80
**12 mM DCA + 250 μM DS**	100	755	780
**12 mM DCA + 1500 μM CU**	100	780	614

[1] Not detected.

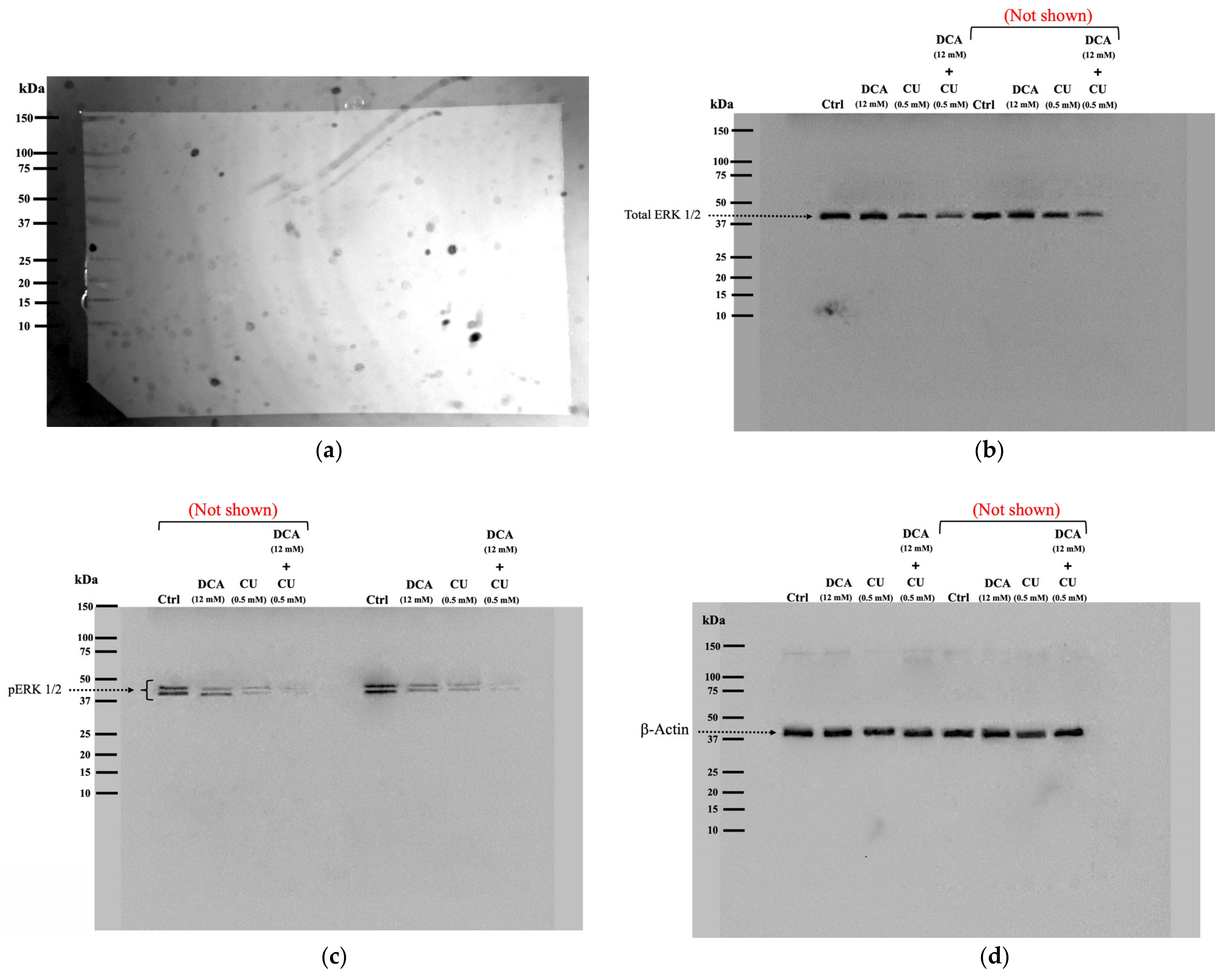

Supplementary Figure S7. Full-length of western blots corresponding to Figure 9. (**a**) Precision Plus Protein™ Kaleidoscope molecular marker as reference, (**b**) uncropped blot from Total ERK 1/2, (**c**) uncropped blot from pERK and (**d**) uncropped blot from β-Actin, used as a loading control.

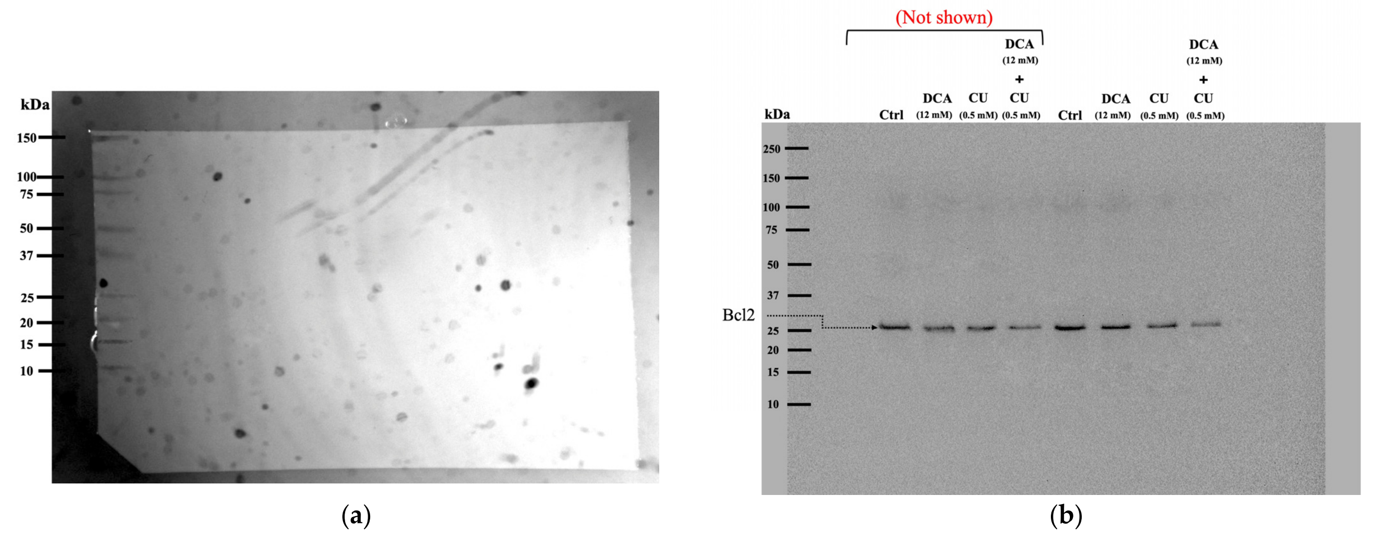

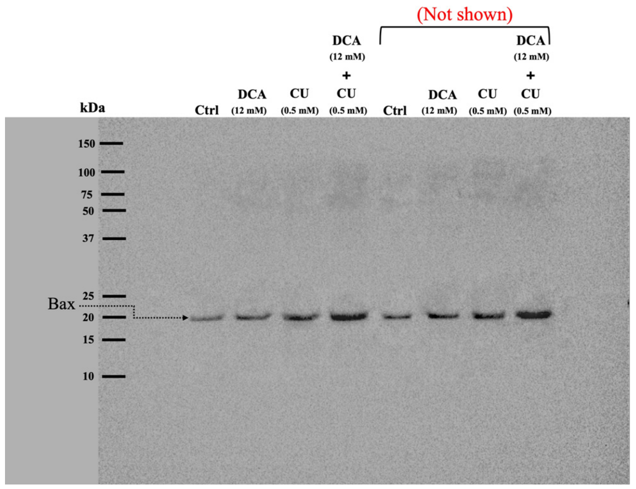

(c)

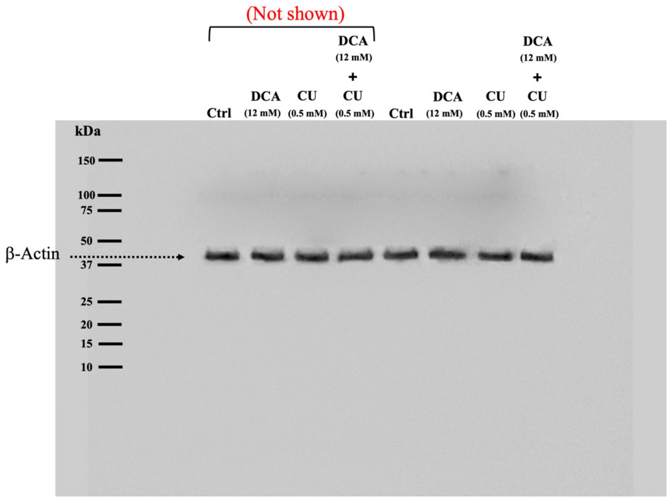

(d)

Supplementary Figure S8. Full-length blots corresponding to Figure 9. (**a**) Precision Plus Protein™ Kaleidoscope molecular marker as reference, (**b**) uncropped blot from Bcl2, (**c**) uncropped blot from Bax and (**d**) uncropped blot from β-Actin, used as a loading control.

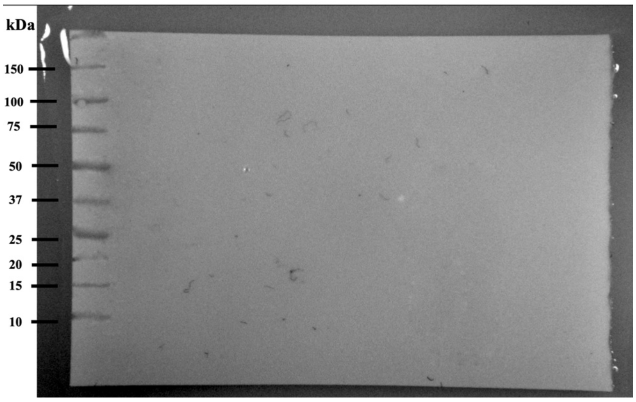

(a)

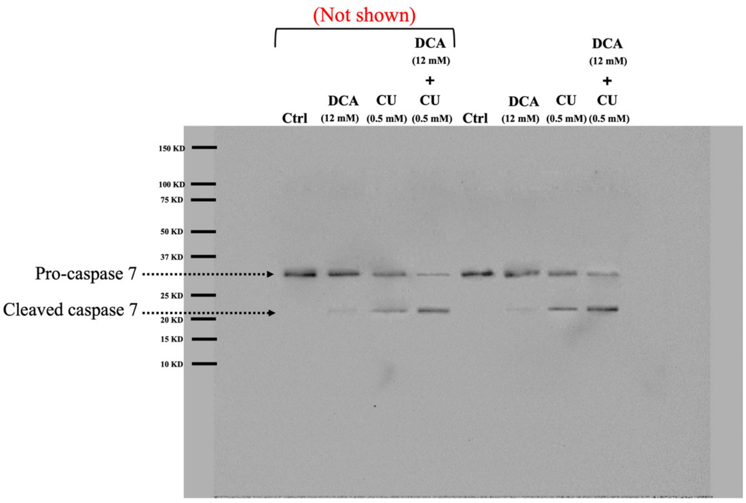

(b)

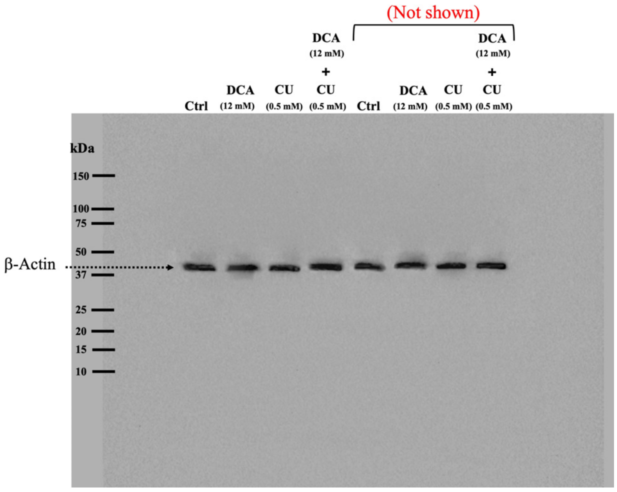

(c)

Supplementary Figure S9. Full-length of western blots corresponding to Figure 9. (**a**) Precision Plus Protein™ Kaleidoscope molecular marker as reference, (**b**) uncropped blot from Procaspase 7 and Caspase 7 cleaved and (**c**) uncropped blot from β-Actin, used as a loading control.

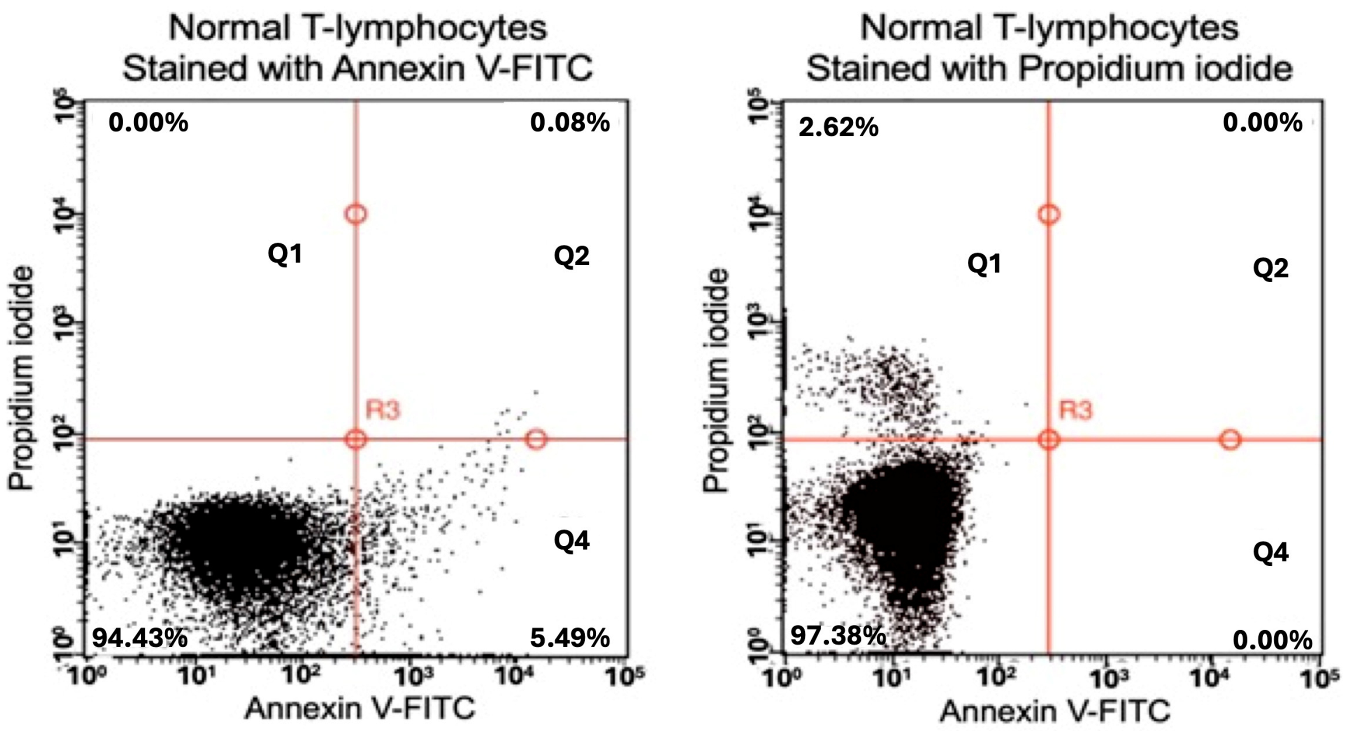

(**a**)

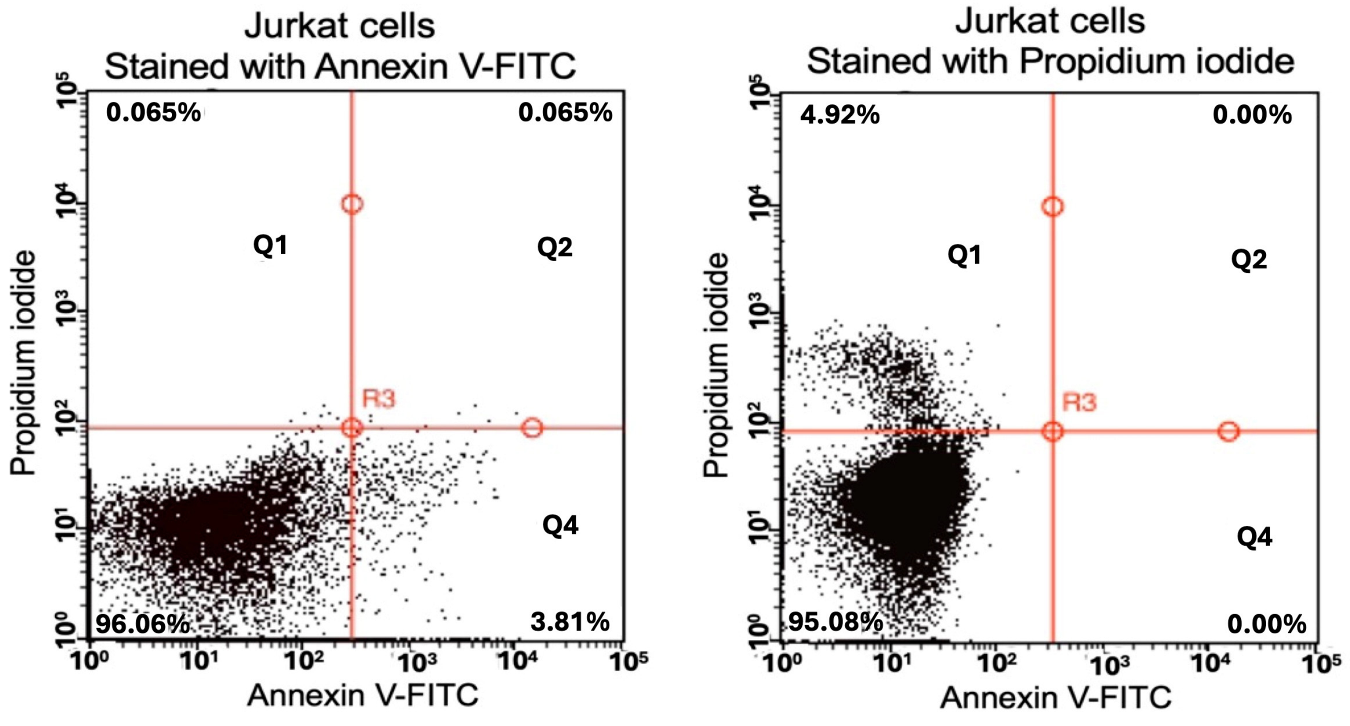

(**b**)

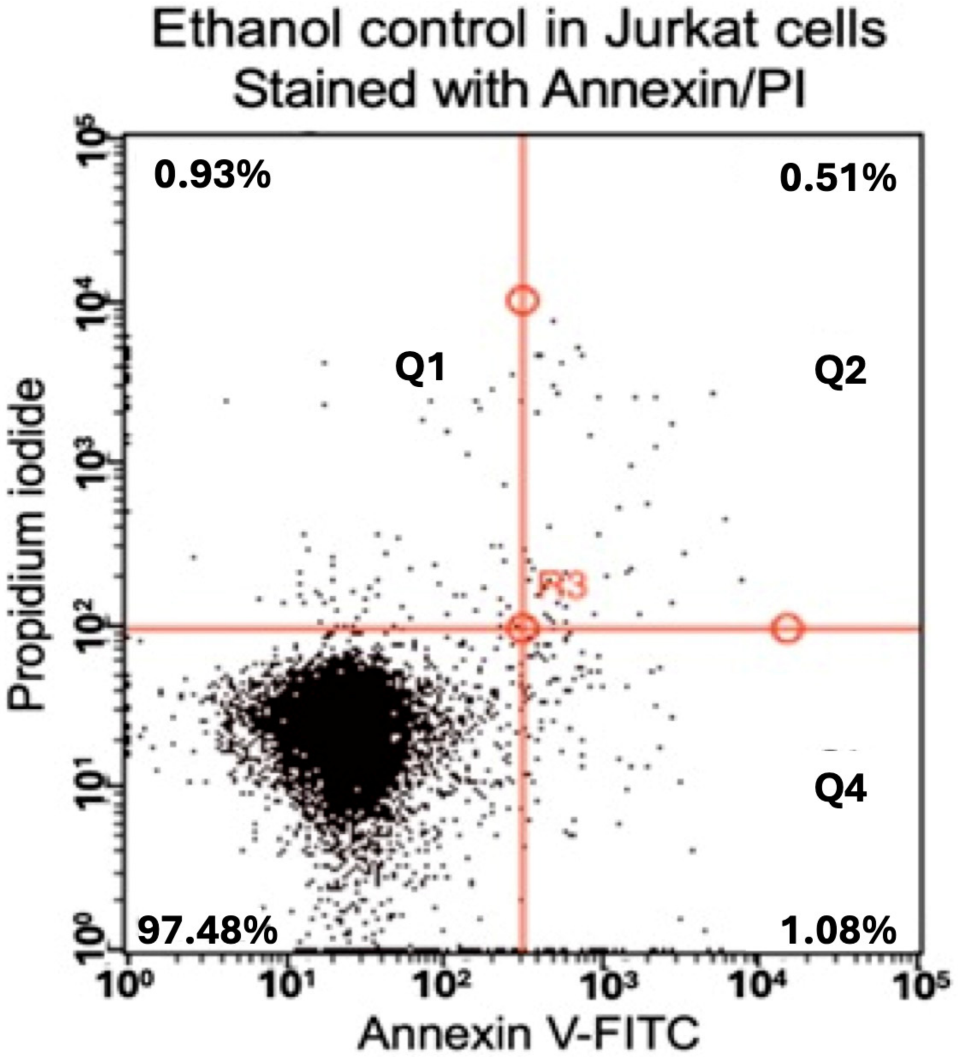

(**c**)

Supplementary Figure S10. Representative image of flow cytometry analysis for apoptosis and necrosis differentiation. To differentiate between apoptotic and necrotic cells, normal T-lymphocytes and Jurkat cells were treated with two concentrations of H_2O_2: 50 μM to induce apoptosis and 500 μM to induce necrosis. After a 5-h incubation at 37 °C, cells were washed, resuspended at a density of 1 × 10^6 cells/mL, and stained with Annexin V and Propidium Iodide for flow cytometry analysis. (**a**) normal T-lymphocytes, (**b**) Jurkat cells and (**c**) Jurkat cells treated with 1.5% ethanol. Q1, necrotic cells; Q2, late apoptotic cells and Q4, early apoptotic cells. The data presented is representative of 100,000 cells analyzed across two independent experiments.

Supplementary Table S5. Flow cytometry analysis of Normal T-Lymphocytes after drug treatment.

Group	Early apoptosis (%)	Late apoptosis (%)	Necrosis (%)	Viability (%)
**Control**	-	-	2.62	97.38
**12 mM DCA**	4.8	2.28	2.33	90.59
**100 μM DS**	6.4	3.2	2.23	88.17

**250 µM CU**	5.92	2.17	2.55	89.36
**DCA + DS**	6.52	5.6	3.63	84.25
**DCA + CU**	6.59	3.95	4.29	85.17

Supplementary Table S6. Flow cytometry analysis of Jurkat cells after drug treatment.

Group	Early apoptosis (%)	Late apoptosis (%)	Necrosis (%)	Viability (%)
**Control**	1.08	0.265	0.595	98.06
**12 mM DCA**	3.09	4.7	11.09	81.12
**100 µM DS**	5.13	10.63	9.67	74.57
**250 µM CU**	3.28	10.11	9.15	77.46
**DCA + DS**	8.13	19.54	15.49	56.84
**DCA + CU**	3.38	21.38	16.54	58.7

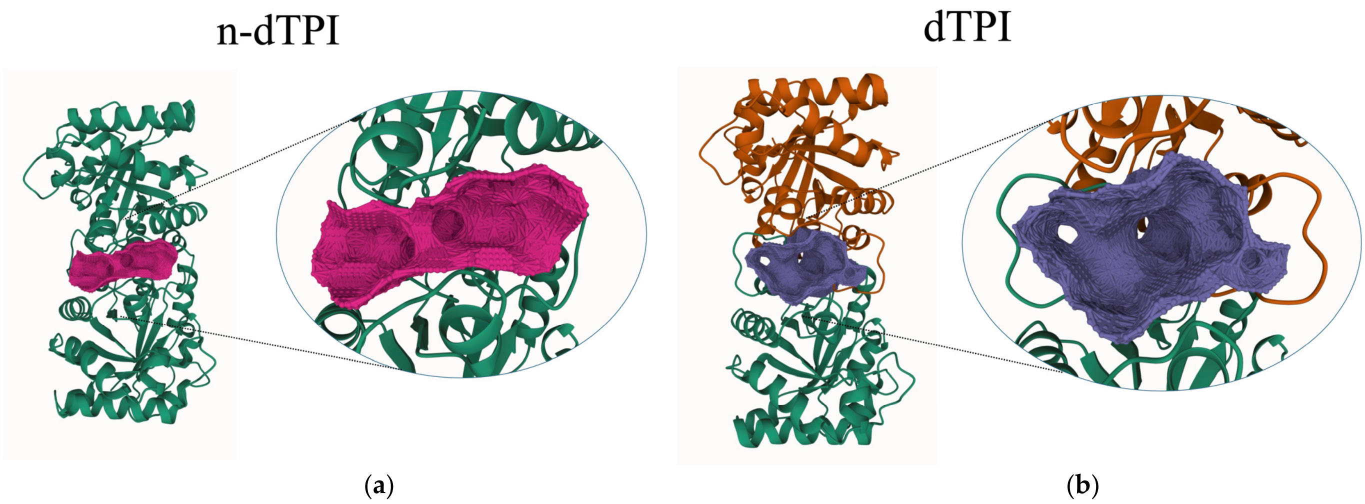

Supplementary Figure S11. Secondary structure representation of TPI dimer. The secondary structure of the TPI is shown in a loop and ribbon format for both subunits within the homodimer. Each panel includes a zoomed inset that emphasizes the dimensional characteristics of the cavity located at the protein-protein interface.

Supplementary Table S7. *In silico* druggability assessment of TPIs.

TPI (PDB ID)	Pred Max pKd	Pred Ave pKd	DrugScore	Druggability assessment
**n-dTPI (2jk2)**	9.18	5.76	-307.00	Weak
**dTPI (4unk)**	10.96	6.97	1181.00	Strong
